# SPOUSAL EDUCATION AND COGNITIVE FUNCTION: EVIDENCE FROM THE ENGLISH LONGITUDINAL STUDY OF AGEING

**DOI:** 10.1093/geroni/igae098.2149

**Published:** 2024-12-31

**Authors:** Hui Liu, Juwen Wang, Wencheng Zhang, Callie Zaborenko

**Affiliations:** Purdue University, West Lafayette, Indiana, United States; Purdue University, West Lafayette, Indiana, United States; Purdue University, West Lafayette, Indiana, United States; Purdue University, West Lafayette, Indiana, United States

## Abstract

Education is one of the strongest social factors influencing cognitive outcomes. Previous studies have predominantly focused on the impact of one’s own education, with less attention given to the education of one’s spouse. Guided by the “linked lives” perspective, this study examines the association between spousal education and cognitive function among older couples in England. We analyzed data from the English Longitudinal Study of Ageing (ELSA) Waves 7-9, 2014-2019. The sample comprised 7,131 married and cohabiting couples. Cognitive function was assessed using the modified version of the Telephone Interview for Cognitive Status (TICS). Results from the Actor-Partner Interdependence Model (APIM) showed a significant correlation in cognitive trajectories between spouses within dyads. Cross-sectional APIM analysis revealed that higher educational attainment of both spouses was associated with better cognitive function in both themselves and their partners. Longitudinal APIM growth curve analysis suggested that the association between spousal education and cognitive function was mainly driven by correlations at the initial levels, with no significant association observed with the slope of cognitive trajectories over time. We discuss these findings in the context of England and compare them to recent studies conducted in the U.S. and Mexico. Potential explanations for both similarities and differences in findings across studies are explored, shedding light on the nuanced interplay between spousal education and cognitive function.

